# Gruber on Ear Diseases
*"A Text-book of the Diseases of the Ear." by Josef Gruber, M.D., Professor of Otology in the Imperial Royal University of Vienna, &c. Translated from the second German edition by Edward Law, M.D., and Coleman Jewell, M.B. (580 pp.) (London: H. K. Lewis.)

**Published:** 1891-01-17

**Authors:** 


### THE PRACTITIONERS' BOOKSHELF.

### GRUBER ON EAR DISEASES.*

Professor Gruber's work on "Diseases of the Ear" has hitherto been accessible only to comparatively few English and American medical men who are conversant with the German language, and Drs. Law and Coleman have done a good service in translating the second German edition into English.

The classification of the matter in this work is to be commended highly. The first part, occupying about one hundred pages, is devoted to the anatomical and physiological description of the organ of hearing, and this is probably the most accurate and complete anatomical description of the ear that has yet been published. "Without accurate anatomical knowledge the successful study of otology is impossible." By this arrangement also the further consideration of the normal anatomical condition of the parts in each region, which would have necessarily involved frequent repetitions, is avoided.

Part II. contains full directions for the examination of aural cases, and a description of the various methods adopted in their observation.

The third, or special part, naturally occupying the greater part of the volume is devoted to the recognition and treatment of diseases of the ear, and it is characteristically excellent throughout.

An individual experience based on the treatment of nearly 35,000 cases of ear diseases in the Vienna General Hospital might well form the ground work of an exposition of Professor Gruber's personal views in otology; but this work is rendered much more valuable to the practitioner by the very fair and full treatment of the methods of practice of otologists generally, and the extensive experience and discrimination of the distinguished author has enabled him to very fairly estimate the relative value of these theories and practices.

We venture to think, however, that Professor Gruber overestimates the disadvantages of Politzer's method of inflating the eustachian tubes, and that his method of making the patient forcibly pronounce hak, hek, hik, hok, huk, cannot be compared with the former for efficiency; for although "the swallowing of water, with its attendant unpleasantness is avoided" it rarely succeeds, and in those exceptional and rare cases where it is undesirable to cautiously employ Politzer's method, the Kalsalvan process is to be preferred.

A distinctive feature of the work is the uniform excellence of the illustrations which are very numerous, and the coloured figures in the plates "form, indeed, a complete atlas of the appearances exhibited by the tympanic membrane in different pathological conditions." The translation "while faithfully following the German text," is expressed in good idiomatic English. The book is printed in large clear type on good paper, and the work of the printers is in keeping with the high standard of its contents.

To the specialist this treatise is essential, while for the general practitioner no work on otology is more to be recommended.

* "A Text-book of the Diseases of the Ear," by Josef Gruber, M.D., Professor of Otology in the Imperial Royal University of Vienna, &c. Translated from the second German edition by Edward Law, M.D., and Coleman Jewell, M.B. (580 pp.) (London: H. K. Lewis.)

